# Empirical Consequences of Current Recommendations for the Design and Interpretation of Noninferiority Trials

**DOI:** 10.1007/s11606-017-4161-4

**Published:** 2017-09-05

**Authors:** Scott K. Aberegg, Andrew M. Hersh, Matthew H. Samore

**Affiliations:** 1 Pulmonary Division, University of Utah School of Medicine, 30 N 1900 E, 701 Wintrobe, Salt Lake City, UT 84132 USA; 2 Division of Epidemiology, University of Utah School of Medicine, 30 N 1900 E, Salt Lake City, UT 84108 USA

## Abstract

**Background:**

Noninferiority trials are increasingly common, though they have less standardized designs and their interpretation is less familiar to clinicians than superiority trials.

**Objective:**

To empirically evaluate a cohort of noninferiority trials to determine 1) their interpretation as recommended by CONSORT, 2) choice of alpha threshold and its sidedness, and 3) differences between methods of analysis such as intention-to-treat and per-protocol.

**Design:**

We searched MEDLINE for parallel-group randomized controlled noninferiority trials published in the five highest-impact general medical journals between 2011 and 2016.

**Main Measures:**

Data abstracted included trial design parameters, results, and interpretation of results based on CONSORT recommendations.

**Key Results:**

One hundred sixty-three trials and 182 noninferiority comparisons were included in our analysis. Based on CONSORT-recommended interpretation, 79% of experimental therapies met criteria for noninferiority, 13% met criteria for superiority, 20% were declared inconclusive, and 2% met criteria for inferiority. However, for 12% of trials, the experimental therapy was statistically significantly worse than the active control, but CONSORT recommended an interpretation of inconclusive or noninferior. A two-sided alpha equivalent of greater than 0.05 was used in 34% of the trials, and in five of these trials, the use of a standard two-sided alpha of 0.05 led to changes in the interpretation of results that disfavored the experimental therapy. In four of the five comparisons where different methods of analysis (e.g., intention-to-treat and per-protocol) yielded different results, the intention-to-treat analysis was the more conservative. In 11% of trials, a secondary advantage of the new therapy was neither reported nor could it be inferred by reviewers.

**Conclusions:**

In this cohort, the design and interpretation of noninferiority trials led to significant and systematic bias in favor of the experimental therapy. Clinicians should exercise caution when interpreting these trials. Future trials may be more reliable if design parameters are standardized.

## INTRODUCTION

Noninferiority trials are used to compare a new therapy (NT) to an active control (AC) when the use of a placebo control is not ethically feasible. The prevalence of noninferiority trials is increasing,^1^^,^ 2 despite concerns about their validity owing to several design and interpretation controversies.^3^^,^ 4 Compared to superiority trials, the interpretation of noninferiority trials is less straightforward, and there is greater flexibility and variability in their design parameters. Little empirical research has evaluated how these unique features may affect the validity of noninferiority trial results and conclusions.

**Figure 1:**
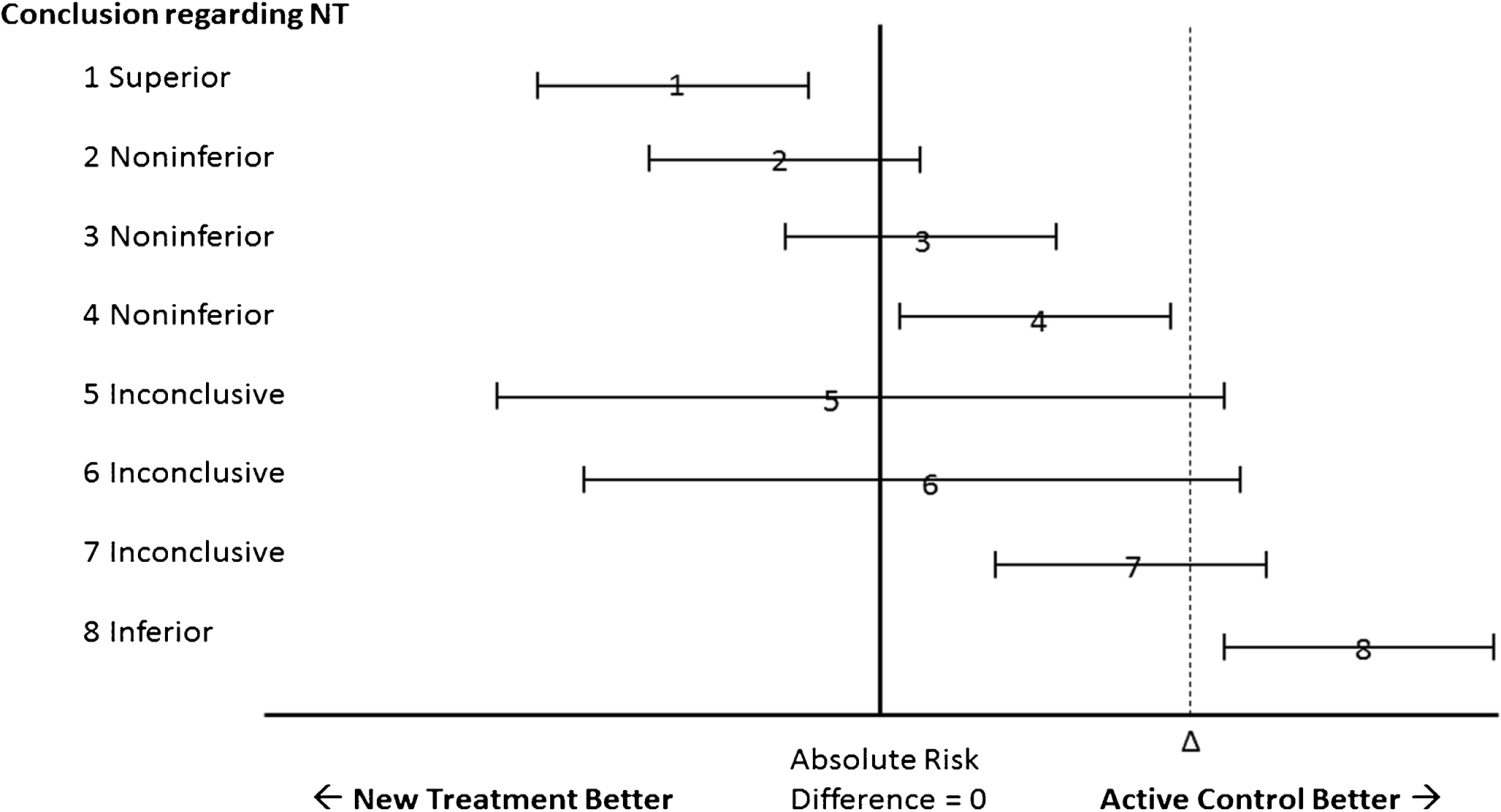
Simulacrum of the CONSORT diagram for interpreting the results of noninferiority trials. According to CONSORT, noninferiority can be declared whenever the upper bound of the confidence interval of the difference between the two therapies does not include delta, as in scenarios 1–4. Whenever the upper bound of the confidence interval exceeds delta, as in scenarios 5–7, noninferiority cannot be declared, because the plausible values of the parameter include some values greater than delta. When both the upper and lower bounds of the confidence interval exceed delta, the NT is declared inferior to the AC, as in scenario 8. Scenario 1 represents all situations in which the upper bound of the confidence interval is less than zero—that is, any statistically significant result favoring the NT garners a declaration of superiority for the NT. By contrast, in scenarios 4 and 7, where there is a statistically significant difference favoring the AC, the NT is not declared inferior in this schematic, but rather noninferior (scenario 4) or inconclusive (scenario 7). NT, new treatment; AC, active control; Δ, delta (the pre-specified margin of noninferiority).

In 2006, the CONSORT [Consolidated Standards of Reporting Trials] Group provided specific recommendations for the reporting of noninferiority and equivalence trials.^5^^,^ 6 Our Figure 1 is a simulacrum of the CONSORT schematic. As explained in the caption, asymmetry in interpretation using this schematic creates a potential bias in favor of the new or experimental therapy.^7^ The frequency with which biased interpretation results from this asymmetry is unknown.

In a superiority trial, a two-sided alpha threshold for statistical significance is conventionally set at <0.05, whereas alpha thresholds in noninferiority trials are more variable.^6^ No investigations to date have evaluated whether the selection of an alpha threshold less stringent than a two-sided 0.05 equivalent influences the conclusions of noninferiority trials.

It is commonly noted that intention-to-treat analyses, recommended for superiority trials, are less conservative than per-protocol or as-treated analyses in noninferiority trials.^6^^,^ 8 ^–^ 11 However, only one small study^12^ has investigated whether the conclusions of noninferiority trials vary by method of analysis, and whether per-protocol and as-treated analyses are more conservative.

Finally, a proposed ethical prerequisite for the use of a noninferiority design states that the NT must have known advantages such as reduced cost, greater convenience, or fewer side effects to justify the randomization of patients to a therapy with unknown efficacy.^13^ This requirement is not met in some noninferiority trials,^14^^,^ 15 but we are not aware of any empirical investigation of the frequency with which this occurs.

Our aim was to investigate how the aforementioned characteristics of noninferiority trials affect their results and the validity of their conclusions.

## METHODS

We searched MEDLINE using the search terms “noninferiority,” “noninferior,” “non-inferiority,” and “non-inferior” combined with the name of the journals as recognized by MEDLINE and published between June 1, 2011, and November 1, 2016. We limited our search to the five highest-impact general medical journals in order to focus on the most widely cited and possibly highest-quality articles.^16^^–^ 18 We reviewed the resulting abstracts to identify articles that met our inclusion criteria: prospective, parallel-group randomized controlled trials where the primary outcome was tested using a noninferiority hypothesis. We then reviewed the full manuscripts and excluded trials that had a cluster-randomized design, trials where the data were incomplete or could not be summarized, those that used a Bayesian methodology, and those that did not use an AC (e.g., Food and Drug Administration [FDA]-mandated placebo-controlled post-approval safety studies). One author (SKA) abstracted the data from the trials into a standardized form and employed a system of redundant checks to ensure data accuracy. Another author (AMH) checked a sample of the data to ensure accuracy. We tabulated basic data about the trial including methodological and statistical design as reported. In trials where more than one analysis was reported (e.g., intention-to-treat [ITT], per-protocol [PP], as-treated [AT]), we determined whether the results differed depending on the analytical method.

We characterized trial results in terms of the point estimate and confidence intervals in accord with Figure 1 of the CONSORT statement, using a 95% two-sided confidence interval, calculated from the raw numbers provided in the manuscript, and coded the trial authors’ conclusions regarding declarations of noninferiority, superiority, inferiority, or inconclusive results, and whether they differed from CONSORT. We used the first outcome mentioned in the manuscript as the primary outcome for our data set when multiple outcomes were reported. Some trials compared multiple interventions, e.g., multiple doses of a new drug against one AC group, and we considered these to represent separate comparisons. In determining whether justification for the selection of a delta value was presented, we coded trials as having “none” if no mention whatsoever was made as to how it was selected, “abstract” if some mention was made but it was vague or irreproducible, and “concrete” if an explicit reproducible justification was provided.

In determining whether the NT had advantages that justified its evaluation using a noninferiority design, we coded trials as having “none” if no advantages were mentioned nor could they be inferred, “inferred” if none were reported but they could be inferred, and “explicit” if the purported advantages of the NT were explicitly stated in the manuscript. Because we were interested in the numerical statistical design features of the trials, we did not perform a subjective quality analysis as is done in a traditional meta-analysis. Likewise, because of highly variable reporting of sponsor and funding sources, and the investigators’ independence from them or lack thereof, we did not abstract these data.

Summary statistics are presented for abstracted values. Chi-square and Student’s *t* tests were used for exploratory analyses. Stata version 14 software (StataCorp LP, College Station, TX, USA) was used for all analyses, including recalculation of two-sided 95% confidence intervals.

## RESULTS

**Figure 2:**
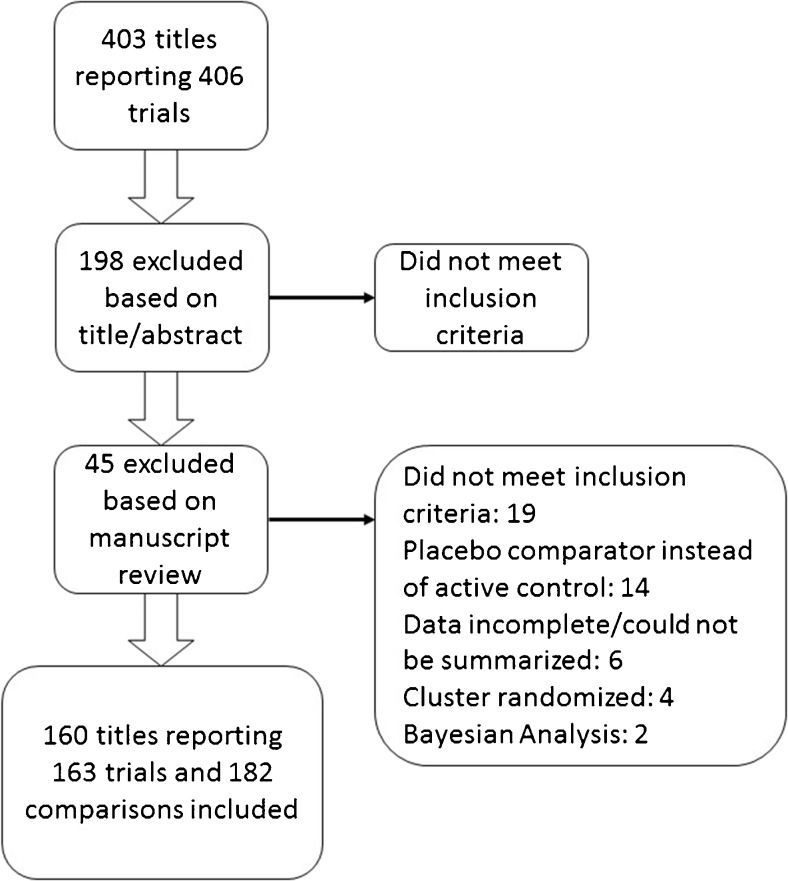
Flow diagram showing the results of our search.

Figure 2 shows the results of our search; 160 included manuscripts reported the results of 163 distinct trials and 182 noninferiority comparisons reported for those trials.

Table 1 shows the characteristics of the trials. The majority of trials (78%) were published in the two highest-impact journals. Therapies pertaining to five specialties (infectious diseases, hematology/oncology, cardiology, pulmonary/critical care, and endocrinology) accounted for 91% of all trials in our cohort. Reporting was complete (100%), with no missing data for the primary outcome, delta, power, alpha sidedness, and planned sample size. Reporting of alpha was complete in 98.5% of trials, and the primary method of analysis was reported in 95.5%. Justification for delta, the pre-specified margin of noninferiority, was not reported in 58% of trials, and in only 25% was reported in a way that allowed replicability of its method of determination. Almost one-third of the trials (32%) used a two-sided alpha equivalent greater than the conventional standard for superiority trials (0.05), with four trials using the equivalent of a two-sided alpha of 0.20.

**Table 1:** Characteristics of the Trials in Our Cohort

		No. (%)
Journal	NEJM	64 (39%)
	Lancet	63 (39%)
	JAMA	23 (14%)
	BMJ	8 (5%)
	Annals	5 (3%)
Year	June–December 2011	12 (7%)
	2012	25 (15%)
	2013	34 (21%)
	2014	22 (14%)
	2015	43 (26%)
	January–October 2016	27 (17%)
Top specialties	Infectious diseases	26%
	Hematology/oncology	25%
	Cardiology	17%
	Pulmonary/critical care	15%
	Endocrinology	8%
Primary outcome measure (*n* = 163 trials)	Absolute risk difference	114 (70%)
	Mean	26 (16%)
	Hazard ratio	13 (8%)
	Relative risk difference	8 (5%)
	Odds ratio	2 (1%)
Primary analysis (*n* = 163 trials)	Intention-to-treat	95 (58%)
	Modified intention-to-treat	36 (22%)
	Per-protocol	24 (15%)
	As treated	2 (1%)
	Not reported	6 (4%)
Secondary analysis (*n* = 163 trials)	Intention-to-treat	14 (9%)
	Modified intention-to-treat	7 (4%)
	Per-protocol	79 (50%)
	As treated	10 (6%)
	None	48 (30%)
Outcome is or includes mortality (*n* = 163 trials)		49 (30%)
Delta for comparisons with proportional outcome measure (*n* = 137 trials)	Mean delta	0.087
	Lowest value	0.004
	Highest value	0.25
	Mean delta for outcomes that do not include mortality (*n* = 89)	0.1
	Lowest value for outcomes that do not include mortality	0.0057
	Highest value for outcomes that do not include mortality	0.25
	Mean delta for outcomes that include mortality (*n* = 48)	0.061
	Lowest value for outcomes that include mortality	0.004
	Highest value for outcomes that include mortality	0.19
Delta justification (*n* = 163 trials)	Not reported	95 (58%)
	Vague and non-reproducible	27 (17%)
	Concrete and reproducible	41 (25%)
Alpha, one-sided equivalent (*n* = 163 trials)	≤ 0.025	105 (66%)
	0.05	51 (32%)
	0.1	4 (2.5%)
	Not reported	3 (2%)
Two-sided hypothesis test (*n* = 163 trials)		62 (38%)
CONSORT confidence interval categorization (*n* = 183 comparisons)	1 (new treatment superior)	28 (15%)
	2 (new treatment noninferior)	67 (37%)
	3 (new treatment noninferior)	46 (25%)
	4 (new treatment noninferior, but old treatment statistically better by less than delta)	3 (2%)
	5 (inconclusive)	0 (0%)
	6 (inconclusive)	19 (10%)
	7 (inconclusive, but old treatment statistically better, by less than delta)	15 (8%)
	8 (new treatment inferior)	4 (2%)
Advantage of new therapy	Explicitly stated	114 (70%)
	Could be inferred	31 (19%)
	Neither stated nor able to be inferred	18 (11%)

NEJM, *New England Journal of Medicine*; Annals, *Annals of Internal Medicine*

For trials that used primary outcomes convertible to an absolute risk difference (*n* = 137), the mean pre-specified delta was 8.7%, with a range of 0.4% to 25%. For trials where mortality was the primary outcome or part thereof (*n* = 48), the mean pre-specified delta was 6.1%, with a range of 0.4% to 19.1%, and if mortality was not part of the outcome (*n* = 89), the mean pre-specified delta was 10.0%, with a range of 0.57% to 25%. The mean observed delta for the primary outcome in the 151 comparisons convertible to an absolute risk difference was +0.04% (range − 36.6% to +26.9%), with 61 point estimates favoring NT and 63 favoring AC. Of the total 182 comparisons, 95 point estimates favored the new treatment and 87 favored the alternative.

Among all 182 comparisons, 28 (15%) were categorized as demonstrating the new treatment to be superior (scenario 1); 113 (62%) were classified as noninferior (scenario 2 or 3); three (2%) were found to show statistically significant evidence that the new treatment was worse but were considered noninferior (scenario 4); 19 (10%) were inconclusive (scenario 5 or 6); in 15 (8%) the new treatment was statistically significantly worse, but the result was considered inconclusive (scenario 7); and four (2.0%) found the new treatment to be inferior (scenario 8). In total, there were 28 statistically significant results favoring the NT (scenario 1), and 22 statistically significant results favoring the comparator therapy (scenarios 4, 7, and 8 combined; difference not significant). Table 2 lists the trials where a scenario 4 or 7 result was obtained.

**Table 2:** Trials with Statistically Significant Results Disfavoring the New Therapy but Not Considering It Inferior

First author	Disease	New therapy	Active control	Outcome	Delta	Result (95% CI)
Roberts^19^	Pediatric respiratory failure	High-flow nasal cannula	CPAP	Tx failure	0.1	0.122 (0.06–0.19)
Geisler^20^	Chlamydia infection	Azithromycin	Doxycycline	Tx failure	0.05	0.032 (0.004–0.06)
Kaul^21^*	Coronary disease	Paclitaxel stent	Everolimus stent	Composite, including death	0.04	0.027 (0.01–0.05)
Gillespie^22^	Tuberculosis	Moxifloxacin replaces ethambutol	Standard TB Tx	Tx failure	0.06	0.07 (0.03–0.11)
Bwakura-Danbarembizi^23^	HIV	Stopping SMX prophylaxis	Continued SMX prophylaxis	Hospitalization or death	0.03	0.06 (0.01–0.11)
Stevenson^24^	Rectal cancer	Laparoscopic surgery	Open surgery	Pathological outcomes	0.08	0.07 (0.01–0.13)
Salminen^25^^†^	Appendicitis	Antibiotics	Surgery	Tx failure	0.24	0.27 (0.22–0.33)
Hooton^26^	Urinary tract infection	Cefpodoxime	Ciprofloxacin	Clinical cure	0.1	0.11 (0.03–0.18)
Bachelez^27^	Rheumatoid arthritis	Tofacitinib 5 mg dose	Etanercept	PASI75 score	0.15	0.19 (0.12–0.27)
Behringer^28^	Hodgkin’s lymphoma	ABV	ABVD	Tx failure	0.06	0.12 (0.06–0.18)
Behringer^28^	Hodgkin’s lymphoma	AVD	ABVD	Tx failure	0.06	0.04 (0.01–0.07)
Vaidya^29^^‡^	Breast cancer	Targeted XRT	Whole breast XRT	Local recurrence	0.025	0.007 (0.0004–0.014)
Buse^30^	Diabetes	Exenatide	Liraglutide	Mean change in glycated hemoglobin	0.25%	0.0021 (0.0008–0.0033)
Lindson-Hawley^31^	Smoking	Gradual cessation	Abrupt cessation	Smoking cessation	0.095	0.10 (0.03–0.17)
Perkins^32^	ACLS training	E-learning	In-person training	Pass rate	0.05	0.06 (0.03–0.09)
Mol^33^	Venous thrombosis	1-year TED hose	2-year TED hose	Post-thrombotic syndrome rate	0.1	0.07 (0.01–0.13)
Gallwitz^34^^‡^	Diabetes	Linagliptin	Glimepiride	Mean change in glycated hemoglobin	0.35%	0.2 (0.09–0.30)
Fishbane^35^^‡^	Renal failure	Peginesatide	Epoetin	Mean change in hemoglobin	1	0.15 (0.01–0.30)

These trials are categorized as scenarios 4 and 7 based on the Figure 1 diagram. Abbreviations: Tx, treatment; CPAP, continuous positive airway pressure; XRT, X-ray therapy; SMX, sulfamethoxazole; ACLS, advanced cardiac life support

*The authors state in the introduction that paclitaxel stents are inferior to everolimus stents in most patients; in an apparent post hoc analysis, they reverse the noninferiority hypothesis and declare everolimus stents superior to paclitaxel stents, a conclusion that they acknowledged was known before the trial was conducted

^†^ Use of a one-sided 95% CI with an upper bound of 0.32 allowed the authors to correctly classify the result as “inconclusive”

^‡^ One of three CONSORT 4 results where the entire 95% CI lies between 0 and Δ, and the result is considered noninferior

**Figure 3:**
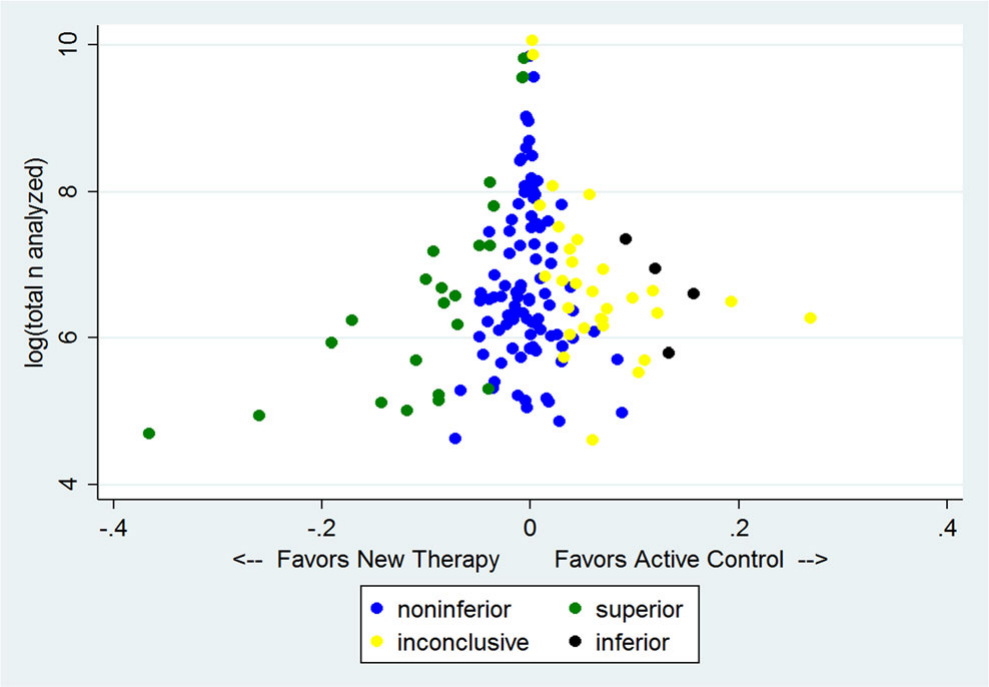
Plot of 151 comparisons of absolute risk differences as a function of the log of the total number of patients analyzed in the trial, color coded by the interpretation of the results as recommended by CONSORT. See text for details.

**Figure 4:**
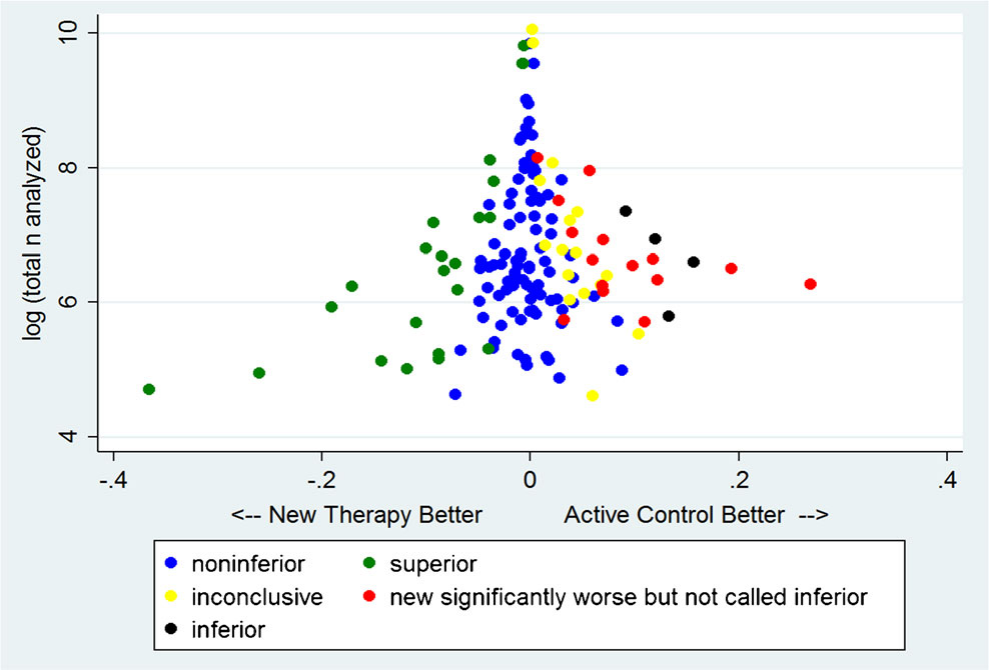
Plot of 151 comparisons with a calculable absolute risk difference as in Figure 3, but with statistically significant results in favor of active control (AC), coded as inconclusive or noninferior in Figure 3, denoted by red in Figure 4.

Figure 3 shows the log of the number of patients analyzed in each trial plotted against the observed result as an absolute risk difference (ARD) for 151 comparisons where an ARD could be calculated. Our results, following CONSORT recommendations, are color coded such that blue dots represent noninferiority, green superiority, yellow inconclusive results, and black inferiority of the NT. There is a paucity of data points on the bottom right of the figure where small trials show large differences favoring the AC, a finding that suggests publication bias. However, formal tests of publication bias (Begg^36^ and Harbord^37^), which are known to be insensitive, were not statistically significant. Figure 4 shows the same data, but here, statistically significant differences favoring AC which were previously coded as inconclusive (CONSORT 7) or noninferior (CONSORT 4) are coded as red. Figure 4 shows that there is a nearly symmetrical distribution of point estimates around a difference of zero. In this figure, there are 23 green data points, and a combined 19 red and black data points, representing a similar distribution of statistically significant results favoring NT and AC, respectively.

In five of the comparisons where the authors used an alpha threshold less stringent than a two-sided 0.05 equivalent, our use of a two-sided 95% confidence interval changed the conclusions. In each of these cases, the more stringent confidence interval made the result less favorable for the NT. In one trial, use of a two-sided 95% confidence interval changed the result from superior to noninferior.^38^ In three trials,^20^^,^ 24 ^,^ 25 the use of a two-sided 95% confidence interval caused a change in the classification of the result from scenario 6 (inconclusive) to scenario 7 (the NT is statistically significantly worse but the result is deemed inconclusive), and in one trial^39^ it changed a scenario 3 (noninferior) to scenario 7 (inconclusive). In all these instances, it was the change from a one-sided to a two-sided confidence interval that changed the categorization of the result, rather than increased stringency from the use of a smaller one-sided alpha value. In the most extreme case (antibiotics vs. appendectomy for acute appendicitis),^25^ the use of a one-sided confidence interval concealed a large and highly statistically significant result in favor of the AC (absolute difference = 27%; 95% CI 21.3–32.9%). Two other trials had statistically significant results favoring the AC by 7% or more that were concealed by the CONSORT interpretative framework.

For eight comparisons, the authors reported conclusions that differed materially from the categorizations recommended by CONSORT. In two trials,^40^^,^ 41 a CONSORT 1 result was obtained, but the authors concluded noninferiority rather than superiority. In two trials,^22^^,^ 28 the authors reported that “noninferiority was not shown” rather than “inferiority was shown.” In three trials where a CONSORT 7 result was obtained (the NT was statistically significantly worse but the result deemed inconclusive), the authors reported that the new treatment was inferior,^42^ stated that the new treatment was statistically worse without concluding inferiority,^19^ or reversed the noninferiority hypothesis and declared the AC superior to the NT.^21^ All eight instances where there was a material difference between the conclusions reported and those recommended by CONSORT involved a statistically significant difference in favor of either AC or NT.

In five of the 117 comparisons where more than one analysis method was reported (e.g., ITT and PP), the conclusion differed depending upon the analysis method. In four^42^^–^ 45 comparisons, the secondary PP analysis was less conservative than the primary analysis, i.e., noninferiority could be claimed with the PP analysis but not the primary analysis. In one trial,^46^ the secondary PP analysis was more conservative and noninferiority criteria were met only with the primary ITT analysis. The authors claimed noninferiority despite this discrepancy.^46^

Among all 163 trials, 70% concretely stated the purported advantage of the NT which would provide ethical justification for randomization with a noninferiority design. In 19% of trials, the advantage was not stated but could be inferred by the reviewers, and in 11% of trials, the advantage of the NT was not stated nor could it be inferred. For more than half of the latter, the NT was a cardiovascular stent device.^15^

## DISCUSSION

In our study of 163 noninferiority trials with 182 noninferiority comparisons published in the five highest-impact general medicine journals during a recent 5-year period, we found that current interpretive recommendations lead to significant systematic and directional biases in the analysis and interpretation of noninferiority trials which almost always favor the experimental therapy. To our knowledge, our analysis is the first to abstract raw data, calculate confidence intervals, categorize the results based on CONSORT, and explore whether and how deviation from the customary design parameters of superiority trials impacts the results and conclusions of noninferiority trials. This is important, since decisions about the use of new, often more expensive therapies increasingly rely on data from noninferiority trials. Most previous analyses of such trials have focused on quality and completeness of reporting of design parameters and results.^17^^,^ 47 We found that statistical design parameters (excluding justification for delta which is perennially deficient) and results were reported with near 100% completeness in the period we studied. However, despite nearly complete reporting, we found that the interpretation of the results of noninferiority trials can hinge critically on both the choice of design parameters and the method of drawing formal conclusions from the results. In our cohort, the use of CONSORT-recommended interpretation with 95% confidence intervals concealed statistically significant results that disfavored the NT in a substantial number of cases. The use of one-sided confidence intervals also concealed statistically significant results disfavoring the NT in a small number of comparisons. Contrary to the recommended preferential use of PP or AT analyses over ITT analyses, we found that the method of analysis seldom affected the results, and when it did, the ITT analysis was more conservative in four out of five trials. While the issues we describe each affect a minority of the trials we analyzed, the collective effect is substantial.

**Figure 5:**
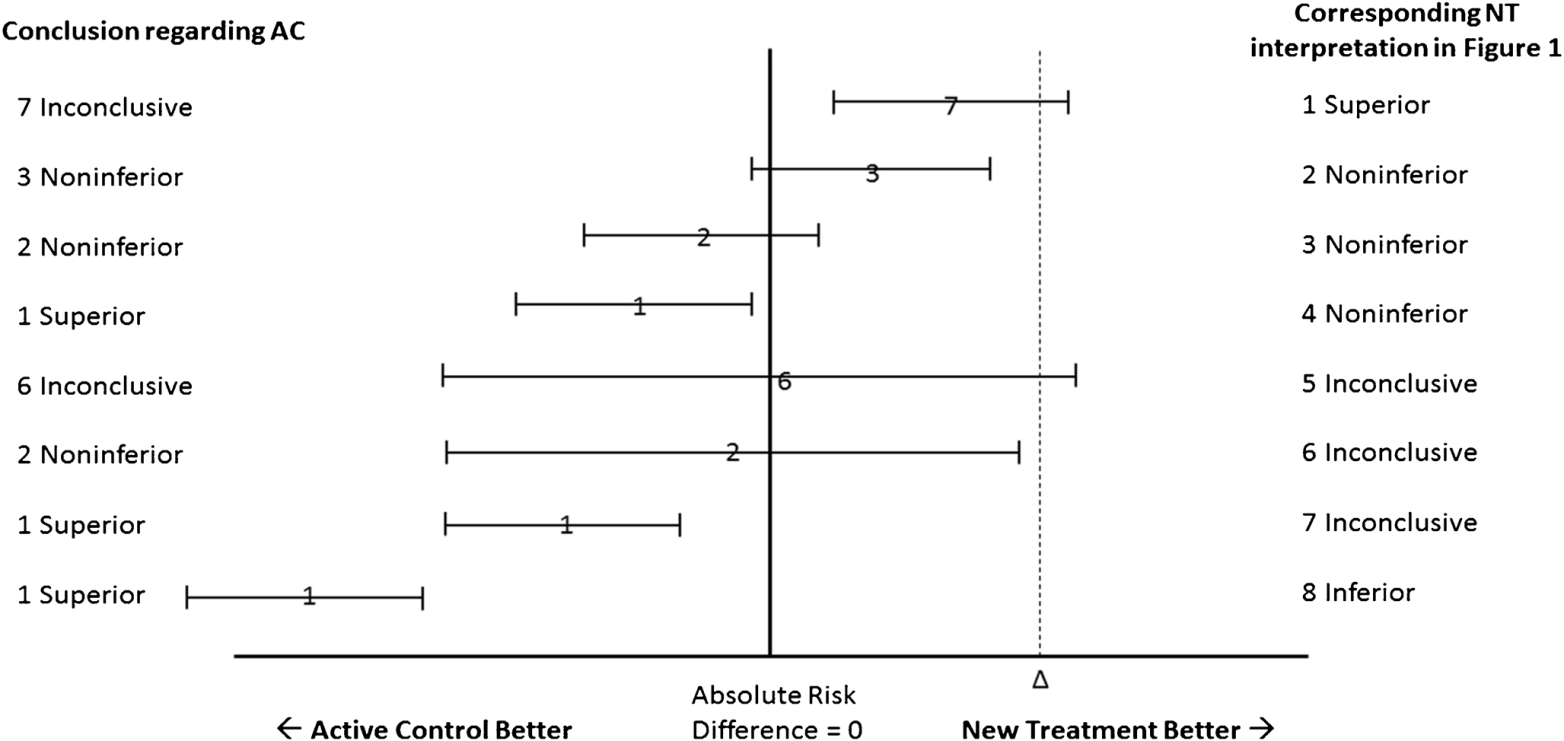
The effect of asymmetrical interpretation of noninferiority results. This schematic shows how the conclusions of a noninferiority trial will differ depending upon which agent, NT or AC, is assigned preferential status on the left of the interpretative diagram. The confidence intervals in this diagram are the mirror images of the confidence intervals in Figure 1, but the NT is now on the right, and AC is on the left (favored side) of the diagram. The absolute risk differences between NT and AC are the same as in Figure 1, and the diagram represents the interpretation that would result if the hypothesis were set up in reverse, to test the noninferiority of AC to NT. The top confidence interval shows a statistically significant difference favoring the new treatment, but instead of a conclusion of superiority (designation 1 in Fig. 1), the result is inconclusive because the upper bound of the confidence interval crosses delta. For the second and third confidence intervals, the conclusion of noninferiority does not change. For the fourth confidence interval, the prior designation 4 (NT is noninferior to AC) becomes a designation 1 (AC superior to NT). The designation of the fifth confidence interval does not change, but the sixth, previously designated inconclusive, becomes a noninferior result for AC. The seventh and eighth confidence intervals, previously showing inconclusive and inferior results for NT, are now designated superior results for AC. Note that the experimental results have not changed—only the assignment of one agent to preferential status on the left of the diagram. Among the eight confidence intervals, four conclusions are materially changed when preferential status is changed.

One of us previously suggested that the use of the CONSORT diagram for the interpretation of the results of noninferiority trials could result in bias.^7^ Our results empirically demonstrate that an asymmetrical interpretation of noninferiority trials creates bias that favors the NT, as illustrated in Figure 5. This figure shows the same results as Figure 1, but as a mirror image, with the AC on the left and the NT on the right of the diagram. As described in the caption, reversing the designation in this way results in a material change to the conclusions in four of the eight scenarios, making the results less favorable for NT. While it has been argued that the designation of NT and AC are not arbitrary because the NT is chronologically “new,”^48^ we found cases in our cohort where the NT was a pre-existing or “old” therapy.^49^^,^ 50 In other trials, placebo was assigned NT status and compared to an unproven treatment as AC,^51^^–^ 56 and in 31 trials, therapies at full intensity were assigned as AC and compared to the same therapies at reduced intensity assigned as NT (Aberegg et al., manuscript under review). Thus, chronology alone appears to be an insufficient criterion for assignment to preferred status as NT. Our results provide the first empirical evidence that this method of interpretation is biased in favor of NT and, in a non-trivial number of trials, effects the concealment of statistically significant results disfavoring NT within the interpretive framework.

Numerous previous reports have examined the adequacy of reporting of noninferiority trials, and have focused consistently on the methodology for the selection of the delta margin.^17^^,^ 47 ^,^ 57 Le Henanff et al.^47^ (2006) reported significant deficiencies in the reporting of statistical design parameters, justification for the delta margin selected (only 20% of reports in their cohort), and reporting of analysis method, with only 43% reporting more than one method (e.g., ITT and PP). Wangge et al.^57^ (2010) found that justification for the delta margin had increased to 46%, but the percentage reporting both types of analysis had not changed. In 2016, Rehal et al.^17^ found that justification for the delta margin remained stubbornly anchored at 46%, with multiple methods of analysis reported in 54%. These reports also described the trial authors’ selections regarding alpha and its sidedness; however, our report is the first to describe and quantify the empirical consequences of these selections. Other investigators have noted the potential advantages of preferential use of ITT analyses in noninferiority trials,^4^^,^ 9 ^,^ 10 but the only other empirical analysis of the ITT/PP difference included just 20 trials and found results that comport with ours, with the ITT analysis being more conservative.^12^

We found that point estimates from trials in our cohort were nearly symmetrically distributed around a difference of zero, similar to two prior investigations.^18^^,^ 58 A large analysis of superiority trials found a similar symmetrical distribution in new versus established treatments.^59^ While the stochastic nature of these results is of epistemological interest and open to speculative interpretation, we reason that this pattern provides support for the idea that new treatments, on average, are not substantially better than existing treatments. On this basis alone, the allowance of preferential treatment for NT is unjustified, especially given our finding that for many NTs, a specific secondary advantage of the NT was not stated and could not be inferred.^14^^,^ 15

Our results have significant implications for the design and analysis of future noninferiority trials. The use of the CONSORT diagram (Fig. 1) for the classification of results should be reevaluated given its obfuscation of results disfavoring NT by a statistically significant margin in 12% of trials in our cohort. This view is bolstered by the fact that the authors of these reports had various ways of drawing conclusions in these CONSORT 4 and 7 scenarios, and that their conclusions often differed from the recommendations. An alternative approach would be to conclude superiority of any treatment with a statistically significant difference favoring it regardless of direction or size. This would remedy much of the directional bias resulting from asymmetry seen in Figure 5. Alternatively, the noninferiority design could be abandoned in favor of equivalence trials with symmetrical delta margins on both sides of unity.^4^^,^ 60

Our finding that there are rarely differences between ITT and PP analyses, and that ITT is usually more conservative when differences arise, suggests that the recommendation to preferentially use PP or AT analyses, which can defeat randomization, should be reevaluated.^4^^,^ 9 ^,^ 10 Our results also confirm that one-sided confidence intervals can sometimes conceal large^25^ and statistically significant differences in outcomes disfavoring the NT. Universal use of two-sided confidence intervals would be preferable. Finally, in addition to an explicit statement justifying the choice of delta, authors of these reports should be required to make an explicit statement about the purported secondary advantages of the NT that form the ethical basis for randomization to an unproven therapy when an effective therapy exists.^3^^,^ 13

Some of the proposed changes to noninferiority trials, such as use of equivalence designs, two-sided confidence intervals, and conservative delta margins, will put upward pressure on sample size in these trials. The implementation of one change could therefore put pressure on another variable upon which sample size depends. Thus, we may expect that the use of more conservative delta values and two-sided confidence intervals could cause investigators to use more lax alpha values or relax power in order to maintain constant sample size.^16^ These compensations could have downstream impacts on the results of future trials. Therefore, it may be most prudent to recommend standardization of statistical design parameters, as is done with superiority trials, and require the uniform use of two-sided 95% confidence intervals in addition to an explicit justification for delta. This standardization would also remove a “researcher degree of freedom”^61^ in the analysis of the results—a very important consideration since pre-registration of noninferiority trials on www.clinicaltrials.gov generally does not include data about statistical design parameters.

A major strength of the present work is that it was a hypothesis-driven^7^ descriptive study to evaluate the empirical consequences of several design recommendations within a theoretical framework. We surveyed a substantial publication epoch in the five highest-impact general medical journals which should capture important noninferiority trials published during that time. Our analysis targeted empirical findings that have not heretofore been reported in analyses of noninferiority trials. Limitations include the selection of only five journals over a 5.5-year period to make our data set manageable, given the labor-intensive nature of our data extraction and recalculation of 95% confidence intervals. Future studies could attempt to confirm our results in a broader selection of journals and over an expanded date range. The possibility of publication bias may influence the results, but our data suggest that publication bias, if present, primarily affects small trials with results favoring AC, and if this is true, our results may understate problems with these trials. Limiting our analysis to the five highest-impact general medical journals may impact the generalizability of our results, but we reason that the included journals likely publish trials of the highest methodological quality, which would cause our results to be an understatement of problems with noninferiority trials.

## Conclusions

The current design and interpretation of noninferiority trials can lead to conclusions which are biased in favor of the therapy designated as new. Clinicians utilizing this literature should cautiously inspect the actual results rather than rely on authors’ interpretations and conclusions. Future noninferiority trials may benefit from standardized design parameters, as is currently customary with superiority trials.
